# Adolescent depression, early psychiatric comorbidities, and adulthood welfare burden: a 25-year longitudinal cohort study

**DOI:** 10.1007/s00127-021-02056-2

**Published:** 2021-03-14

**Authors:** Iman Alaie, Richard Ssegonja, Anna Philipson, Anne-Liis von Knorring, Margareta Möller, Lars von Knorring, Mia Ramklint, Hannes Bohman, Inna Feldman, Lars Hagberg, Ulf Jonsson

**Affiliations:** 1 Department of Neuroscience, Child and Adolescent Psychiatry, Uppsala University, Uppsala, Sweden; 2 Department of Public Health and Caring Sciences, Child Health and Parenting (CHAP), Uppsala University, Uppsala, Sweden; 3 Faculty of Medicine and Health, University Health Care Research Center, Örebro University, Örebro, Sweden; 4 Department of Neuroscience, Psychiatry, Uppsala University, Uppsala, Sweden; 5 Karolinska Institutet Center of Neurodevelopmental Disorders (KIND), Centre for Psychiatry Research, Department of Women’s and Children’s Health, Karolinska Institutet, & Stockholm Health Care Services, Stockholm County Council, Stockholm, Sweden

**Keywords:** Depression, Adolescent, Economic status, Epidemiology, Longitudinal studies

## Abstract

**Purpose:**

Depression at all ages is recognized as a global public health concern, but less is known about the welfare burden following early-life depression. This study aimed to (1) estimate the magnitude of associations between depression in adolescence and social transfer payments in adulthood; and (2) address the impact of major comorbid psychopathology on these associations.

**Methods:**

This is a longitudinal cohort study of 539 participants assessed at age 16–17 using structured diagnostic interviews. An ongoing 25-year follow-up linked the cohort (*n* = 321 depressed; *n* = 218 nondepressed) to nationwide population-based registries. Outcomes included consecutive annual data on social transfer payments due to unemployment, work disability, and public assistance, spanning from age 18 to 40. Parameter estimations used the generalized estimating equations approach.

**Results:**

Adolescent depression was associated with all forms of social transfer payments. The estimated overall payment per person and year was 938 USD (95% CI 551–1326) over and above the amount received by nondepressed controls. Persistent depressive disorder was associated with higher recipiency across all outcomes, whereas the pattern of findings was less clear for subthreshold and episodic major depression. Moreover, depressed adolescents presenting with comorbid anxiety and disruptive behavior disorders evidenced particularly high recipiency, exceeding the nondepressed controls with an estimated 1753 USD (95% CI 887–2620).

**Conclusion:**

Adolescent depression is associated with considerable public expenditures across early-to-middle adulthood, especially for those exposed to chronic/persistent depression and psychiatric comorbidities. This finding suggests that the clinical heterogeneity of early-life depression needs to be considered from a longer-term societal perspective.

**Supplementary Information:**

The online version contains supplementary material available at 10.1007/s00127-021-02056-2.

## Introduction

Depressive disorders are among the leading causes of the burden of disease in both adolescents [1] and adults [2] worldwide. These disorders are common along the lifespan, with a prevalence of major depression estimated to be about 5–6% in adolescence [3], quite similar to the rates reported in adulthood [4]. The first onset is frequently occurring after puberty [5], with an increasing cumulative probability rising from around 5% in early teens to almost 20% in late adolescence [6], and a strong female preponderance (about 2:1) [4, 7]. Adolescent depression is also well known to forecast poor general health [5], mental ill-health [8], and impaired psychosocial functioning [9] in adulthood, although more research is needed to better understand the configuration of risk factors linked to adverse adult outcomes. Importantly, recent meta-analytic findings imply a substantial cost burden of depression at all ages [10]. However, the vast majority of previous studies on the costs of depression have primarily reported on healthcare expenditures among adults [10], and only to a lesser extent on the societal burden of affected adolescents [11].

Considering the overall cost burden, the *indirect* costs constitute by far the greatest component, as reflected in reduced or lost productivity due to unemployment or work disability [12–14]. As a result, governmental or local authorities in certain societies intervene to help individuals meet their basic needs through the provision of financial assistance in the form of unemployment benefits, sick pay, disability pension, or public/social assistance [15, 16]. These ‘hidden costs’ from governmental transfer programs, often called social transfer payments, represent a transfer of purchasing power from general taxpayers to individual recipients, albeit without net increases in the use of resources [17, 18]. As regards the redistribution of economic resources in society, cross-national comparative data suggest that those who are poor, unemployed, or disabled in the Nordic countries tend to be better off than their counterparts in most other parts of the world [15, 19, 20]. One plausible explanation for these differences lies with the advanced welfare regimes in the Nordic countries, whose social policies and systems of support serve to counteract socioeconomic disadvantage and, ultimately, the pervasive effects of poverty among vulnerable groups [16, 21, 22]. While recent large-scale investigations show that people suffering from a mental disorder are facing a larger employment disadvantage, higher disability benefit claims, and also a much larger poverty risk than those without mental disorders [13], there is still a considerable lack of knowledge about how early-life common mental disorders, such as depression, impact on future receipts of welfare benefits. In addition, the few published studies reporting on associations of adolescent depression with adult welfare dependence and unemployment have solely relied on self-reported follow-up data [9], which potentially are biased. Moreover, previous studies have typically focused on exposure to depression in general [8], yet recent research points to a particularly poor prognosis for those with chronic/persistent forms of early depression [23–27]. This implies the need for more detailed and objective data, but also that due consideration should be given to the heterogeneous manifestation of depressive illness, to obtain relevant estimates for decision-making.

In respect of other key features of clinical heterogeneity, the longer-term societal burden of comorbidity in early-onset depression is still poorly understood. Indeed, it is well established that depression, anxiety disorders (ADs), and disruptive behavior disorders (DBDs) co-occur more frequently than would be expected by chance alone [28]. These disorders are all recognized as major causes of disability among older children and adolescents [1, 29], and the adverse adult outcomes of early ADs [30], and DBDs [31], as well as the cost burden of specific DBDs [32], are now well documented. Less is known about the longer-term outcomes following comorbid early-onset depression, as current literature demonstrates mixed findings and even no effects [25, 33–37]. A recent study reported a linear cumulative burden of common childhood mental disorders with regard to adult functional outcomes [38], suggesting the need for a better understanding of early comorbidity and its potentially multiplicative effects over time. Such understanding is central to inform effective allocation of resources, but also to guide prevention and treatment to mitigate long-term impairments [39, 40]. It is, therefore, imperative to have a clear picture of the magnitude of the public expenditures following early-onset depression when also taking major comorbid psychopathology into account. To date, no such estimates are available.

In a recently published study, we reported on the differences in adult earnings between formerly depressed adolescents and nondepressed peers [26]. We found that early chronic/persistent depression, in particular, was linked to lower adult earnings; however, it is unclear how this loss of productivity is reflected in long-term welfare benefits following adolescent depression.

In the present study, the overarching goal was to investigate the associations of adolescent depression with subsequent social transfer payments related to unemployment, work disability, and public assistance. Our aims were (1) to estimate the magnitude of associations between adolescent depression and social transfer payments across early-to-middle adulthood; and (2) to address the impact of major comorbid psychopathology on these associations.

## Methods

### Study population and procedure

This report follows the Strengthening the Reporting of Observational Studies in Epidemiology (STROBE) reporting guidelines for cohort studies [41]. Participants were members of a Swedish community-representative cohort, the Uppsala Longitudinal Adolescent Depression Study (ULADS), which has been prospectively followed from adolescence onwards [42]. The initial epidemiologic investigation comprised a total population of first-year students aged 16–17 in all upper-secondary schools of Uppsala in 1991–1992. In all, 2300 (93% participation rate) underwent screening by means of two self-report questionnaires: the Beck Depression Inventory-Child (BDI-C) [43] and the Center for Epidemiological Studies-Depression Scale for Children (CES-DC) [44]. Adolescents with positive screening, defined as BDI-C ≥ 16 [45] or CES-DC ≥ 30 + BDI-C ≥ 11 [46] or a self-reported suicide attempt, were invited to a blinded structured diagnostic interview. The decision to equate suicide attempt with probable depression was based on the observed high lifetime prevalence of affective disorders among adolescents with suicidal behavior [47–49]. Further, an equal number of adolescents with negative screening, matched for gender, age, and school class, were also invited. Out of 710 selected adolescents, 631 (78% females) participated.

Extensive de-identified data were retrieved in 2017–2018 through linkage of the retained cohort members' unique personal identity number to various nationwide population-based registries kept by government agencies [50]. This 25-year follow-up harvested registry data for 576 (91%) of the original cohort members, excluding 55 individuals who at baseline did not give consent to further participation (*n* = 22), or who subsequently had refused extraction of individualized registry data (*n* = 33). Out of the retained cohort (*n* = 576), only those presenting with an early-onset hypomanic or manic episode (*n* = 37) were excluded for the present study. The rationale for this was that bipolar disorders are classified separately from depressive disorders in the Diagnostic and Statistical Manual of Mental Disorders (DSM) [51], due to key differences in symptomology and etiology. This left a total of 539 eligible cohort members (*n* = 321 with adolescent depression; *n* = 218 nondepressed). A flow chart of the study design is shown in the supplemental information (see Figure S1). Further detailed information about the data collection waves of the ULADS has been reported elsewhere [42]. Ethical approval for the current 25-year follow-up was obtained from the Regional Ethical Review Board in Uppsala (2015/449/1-2).

### Measures

#### Exposure

*Adolescent depression* At baseline, cohort members were assessed with the Diagnostic Interview for Children and Adolescents in the Revised form according to DSM-III-R for Adolescents (DICA-R-A) covering a wide range of childhood and adolescent psychiatric disorders [52]. As the diagnostic assessments were based on DSM-III-R criteria [53], these original classifications were converted to the current DSM-5 taxonomy [51]. The following subgroups were identified:

Persistent depressive disorder (PDD): depressed mood occurring for most of the day, for more days than not, for at least 1 year (*n*=175);

Major depressive disorder (MDD): a current or life-time major depressive episode lasting shorter than 1 year (*n*=82);

Subthreshold depression: a positive screening but no other past or current depressive disorder (*n*=64);

No depression: negative screening, no past or current depressive disorder (*n*=218).

*Comorbidity* As part of the DICA-R-A [52] interviews at baseline, cohort members were also assessed with respect to ADs (separation anxiety, overanxious, and avoidant disorder) and DBDs (oppositional defiant disorder, conduct disorder, and attention-deficit/hyperactivity disorder) of childhood/adolescence. As regards major comorbid psychopathology among the depressed adolescents (*n* = 321), the following subgroups were formed:

Noncomorbid depression (i.e., no ADs or DBDs) (*n*=132);

Depression + ADs (i.e., no DBDs) (*n*=93);

Depression + DBDs (i.e., no ADs) (*n*=44);

Depression + DBDs + ADs (*n*=52).

#### Covariates

In addition to gender, the highest level of education achieved by either parent in 1990 was included as a categorical variable (i.e., tertiary education, yes/no). Information on parental education was retrieved from Statistics Sweden [50]. This was used as a proxy measure of family socioeconomic background, to account for socioeconomic disadvantage [54].

#### Outcomes

Register-based data on social transfer payments were used as main outcome measures. These registry data were retrieved from the Longitudinal Integration Database for Health Insurance and Labor Market Studies (LISA), held by Statistics Sweden [50]. The LISA registry is updated each year by the transmission of annual data on the labor market, educational systems, and social sectors from various source registries.

The outcomes comprised four types of social transfer payments: (1) unemployment benefits, (2) work disability benefits, (3) public assistance, and (4) overall payments combined. These were based on consecutive annual data spanning from 1994 to 2016, covering early (age 18) to middle (age 40) adulthood.

*Unemployment benefits* were defined as individualized transfer payments for those registered as full- or part-time unemployed at the Swedish Public Employment Service [50]. To qualify for unemployment benefits, the individual needs to be unemployed, able to work and ready to accept work that is offered. In addition, the beneficiary must have worked for at least 6 months prior to becoming unemployed. Benefits amount to a capped 80% of lost income, which is further capped after the initial 100 days of unemployment. Unemployment benefits received from participation in active labor market policy programs, serving to support and activate those being outside the labor market, were also included in this outcome. Accordingly, participation in such government-based programs entitles individuals to benefits while being involved in vocational training.

*Work disability benefits* were defined as individualized payments due to sickness absence and disability pension [50, 55]. All residents aged over 16 years who have an income from work or unemployment benefits are eligible for sickness benefits, if there is a reduced work capacity due to disease or injury. For the first 14 days of a sickness period an employee receives sick pay from the employer, though there is one qualifying day (more for self-employed) without any benefits. If the work capacity is still reduced after 14 days, the employee can apply for sickness cash benefits. Sickness allowances, including various sick payments, correspond to approximately 80% of lost income, and presupposes work prior to the benefit claims. Individuals aged 16–64 years, who have a permanently impaired work capacity, are entitled to disability pension. Since 2003, only temporary activity benefits can be granted for young people aged 19–29 years, whereas individuals aged 30–64 can be entitled to permanent sickness compensation. Disability pension amounts to a capped 65% of lost income, with reduced cash benefits for those that have not worked previously or have had a low income.

*Public assistance* was defined as social welfare assistance, housing supplement, housing allowance, and maintenance support. Social welfare assistance is a cash income allowance from the local social authorities, which is given only after a thorough individual means test designed to guarantee people a minimum standard of living [50]. This allowance is designed to cover basic expenditures, such as food and rent, and applies to all individuals in Sweden provided that they try to achieve financial self-sufficiency (i.e., job seeking if unemployed). Housing allowance refers to support for young people aged 18–28 years, and families with children, who need financial help to pay rent or fees for their housing. Housing supplement is a housing allowance for recipients of disability pension, targeting only those eligible for activity benefits or sickness compensation. Maintenance support is an alimony for children with separated parents. In case parents cannot reach an agreement on child support, the residential parent can apply for maintenance support, which is intended to cover costs for the child’s housing, food, clothes, and leisure interests.

*Overall payments combined* were defined as a summed measure of all types of cash benefits and financial assistance described in the aforementioned outcomes.

### Missing data

Across all 23 years of follow-up, 11,814 (95.3%) observations were recorded for each outcome. There were two sources of complete or intermittent missingness in the register-based data: death (*n* = 3) and resettling abroad (*n* = 66). The mean total time residing abroad among those who had emigrated was 7.5 years (SD = 6.1). Cohort members whose parents had missing values on education were assigned a low parental educational level (*n* = 17).

The overall dropout rate did not differ substantially between depressed and nondepressed adolescents. However, those with subthreshold depression were more likely to drop out than other depressed subgroups [42]. As there was a relatively small proportion of missingness, the statistical analyses were based on all available follow-up data.

### Statistical analyses

Longitudinal associations between the exposure and the repeated measures outcome data on social transfer payments were modeled using generalized linear models, with parameters estimated using the generalized estimating equations framework [56]. A series of population-averaged regression models were fitted for each outcome. The regression models made use of an auto-regressive working correlation structure and robust standard errors, chosen based on model fit. All associations were analyzed by fitting a Tweedie distribution with an identity link function to the repeated measures data on social transfer payments, in order to account for the excess of zero counts in each outcome [57]. Throughout all analyses, both unadjusted and adjusted estimates were calculated. The latter included gender and parental education as covariates. Any between-groups differences were estimated as the average of payments per year, with a 95% confidence interval (CI). Statistical significance was set at *p* < 0.05.

The regression models were tested in a series of steps. First, the differences in adult outcomes were analyzed by adolescent depression status, with the nondepressed controls as the reference category. Second, an interaction term was added to examine whether there was a synergistic effect of gender, alongside additional gender-stratified analyses. Third, differences in adult outcomes were analyzed by specific type of depression (i.e., PDD; MDD; subthreshold depression), with the nondepressed controls as the reference. Fourth, differences in adult outcomes were analyzed by the multiple types of comorbid depression (i.e., noncomorbid depression; depression and ADs; depression and DBDs; depression and DBDs and ADs), with the nondepressed controls as the reference. Fifth, differences in overall social transfer payments were also tested in a multivariable analysis including specific type of depression, DBDs, and ADs entered as separate exposure variables. Lastly, additional analyses were performed to look at differences between groups and across time.

The annual payments were converted to the value of January 2019 using Consumer Price Index (CPI) [58]. All payments were converted from Swedish krona (SEK) to US Dollar (USD) using an approximated exchange rate of 1 SEK = 0.1113 USD, as valid in January 2019. All data management and analyses were performed using IBM SPSS Statistics version 26 and STATA version 16.

## Results

Descriptive characteristics of the included cohort (*n* = 539) are summarized in Table 1. About four of five cohort members were females. Among those with adolescent depression (*n* = 321), the majority of cases presented with PDD (*n* = 175, 54.5%). Moreover, a majority of depressed adolescents had some type of early psychiatric comorbidity (*n* = 189, 59%). As regards overall recipiency of social transfer payments, about 12% of nondepressed controls and 4% of depressed adolescents did not receive any payments during the entire follow-up.

**Table 1:** Descriptives of study population

Key characteristics of cohort members	Included cohort (*n* = 539) *n (%)*	No adolescent depression (*n* = 218) *n (%)*	Adolescent depression^a^ (*n* = 321) *n (%)*	Type of adolescent depression		
				Subthreshold depression (*n* = 64) *n (%)*	Major depressive disorder (*n* = 82) *n (%)*	Persistent depressive disorder (*n* = 175) *n (%)*
Females	425 (79%)	171 (78%)	254 (79%)	46 (72%)	68 (83%)	140 (80%)
Low parental education^b^	271 (50%)	104 (48%)	167 (52%)	35 (55%)	50 (61%)	82 (47%)
Other psychiatric disorders of childhood/adolescence						
Anxiety and Disruptive behavior disorders	55 (10%)	3 (1%)	52 (16%)	5 (8%)	9 (11%)	38 (22%)
Disruptive behavior disorders^c^	57 (11%)	13 (6%)	44 (14%)	11 (17%)	11 (13%)	22 (13%)
Anxiety disorders^d^	122 (23%)	29 (13%)	93 (29%)	1 (2%)	25 (31%)	67 (38%)
Noncomorbid depression^e^	132 (24%)	–	132 (41%)	47 (73%)	37 (45%)	48 (27%)
Social transfer payments in adulthood^f^						
Unemployment benefits, M (SD)	666 (2417)	549 (2143)	748 (2589)	635 (2209)	638 (2399)	841 (2792)
Work disability benefits, M (SD)	744 (2993)	503 (2265)	914 (3401)	552 (2965)	848 (3113)	1077 (3659)
Public assistance, M (SD)	345 (1348)	140 (704)	489 (1642)	469 (1625)	480 (1614)	501 (1660)
Overall payments combined, M (SD)	1756 (4114)	1192 (3276)	2151 (4570)	1657 (4023)	1965 (4199)	2419 (4895)
Recipiency of overall payments combined						
0 years	38 (7%)	26 (12%)	12 (4%)	2 (3%)	2 (3%)	8 (5%)
1–2 years	68 (13%)	35 (16%)	33 (10%)	7 (11%)	10 (12%)	16 (9%)
3–5 years	136 (25%)	59 (27%)	77 (24%)	17 (27%)	19 (23%)	41 (24%)
6–10 years	144 (27%)	60 (27%)	84 (26%)	18 (28%)	23 (28%)	43 (25%)
11–15 years	76 (14%)	19 (9%)	57 (18%)	9 (14%)	11 (14%)	37 (21%)
16–23 years	75 (14%)	19 (9%)	56 (18%)	11 (17%)	16 (20%)	29 (17%)

^a^Subthreshold depression, major depressive disorder, and persistent depressive disorder

^b^Neither parent had tertiary education

^c^No anxiety disorder

^d^No disruptive behavior disorder

^e^No anxiety nor disruptive behavior disorder

^f^Average payments received in US dollars per year across ages 18–40

With regard to major comorbid psychopathology, descriptive statistics of cohort members with adolescent depression (*n* = 321) are presented in Table 2.

**Table 2:** Descriptives of cohort members with adolescent depression (*n* = 321)

Characteristics of depressed adolescents	Noncomorbid depression (*n* = 132) *n (%)*	Depression + Anxiety disorders (*n* = 93) *n (%)*	Depression + Disruptive behavior disorders (*n* = 44) *n (%)*	Depression + Disruptive behavior disorders + Anxiety disorders (*n* = 52) *n (%)*
Females	108 (82%)	80 (86%)	30 (68%)	36 (69%)
Low parental education^a^	74 (56%)	41 (44%)	29 (66%)	23 (44%)
Type of adolescent depression				
Persistent depressive disorder	48 (36%)	67 (72%)	22 (50%)	38 (73%)
Major depressive disorder	37 (28%)	25 (27%)	11 (25%)	9 (17%)
Subthreshold depression	47 (36%)	1 (1%)	11 (25%)	5 (10%)
Social transfer payments in adulthood^b^				
Unemployment benefits, M (SD)	628 (2134)	621 (2361)	984 (3235)	1109 (3341)
Work disability benefits, M (SD)	810 (3359)	1055 (3664)	732 (2913)	1062 (3350)
Public assistance, M (SD)	511 (1643)	178 (822)	764 (1878)	784 (2321)
Overall payments combined, M (SD)	1949 (4332)	1854 (4381)	2481 (4798)	2956 (5182)
Recipiency of overall payments combined				
0 years	7 (5%)	4 (4%)	1 (2%)	0 (0%)
1–2 years	19 (14%)	7 (8%)	5 (12%)	2 (4%)
3–5 years	31 (24%)	25 (27%)	12 (28%)	9 (18%)
6–10 years	34 (26%)	27 (29%)	8 (19%)	15 (29%)
11–15 years	18 (14%)	19 (20%)	8 (19%)	12 (24%)
16–23 years	23 (17%)	11 (12%)	9 (21%)	13 (26%)

^a^Neither parent had tertiary education

^b^Average payments received in US dollars per year across ages 18–40

### Associations of adolescent depression with social transfer payments across adulthood

As shown in Table 3, adolescent depression in general was associated with all forms of social transfer payment outcomes, including overall payments, which was estimated as 938 USD (95% CI 551–1326) more per year than the nondepressed controls. Those with PDD evidenced higher recipiency across all outcomes, with overall payments estimated as 1172 USD (95% CI 673–1670) more per year; however, there were less pronounced differences between the other depressed subgroups and the nondepressed controls. In terms of overall payments, there was a higher recipiency among those with MDD (793 USD, 95% CI 176–1410), but this association was nonsignificant among those with subthreshold depression (491 USD, 95% CI − 40 to 1022). Overall, associations remained largely unchanged after covariate adjustment.

**Table 3:** Associations of adolescent depression with social transfer payments across ages 18–40, with nondepressed controls (*n* = 218) as reference

	Adolescent depression^a^ (*n* = 321)		Type of adolescent depression					
			Subthreshold depression (*n* = 64)		Major depressive disorder (*n* = 82)		Persistent depressive disorder (*n* = 175)	
Social transfer payments (USD/year)	Unadjusted	Adjusted^b^	Unadjusted	Adjusted^b^	Unadjusted	Adjusted^b^	Unadjusted	Adjusted^b^
	B (95% CI)	B (95% CI)	B (95% CI)	B (95% CI)	B (95% CI)	B (95% CI)	B (95% CI)	B (95% CI)
Unemployment benefits								
All	202* (21, 384)	173* (2, 343)	108 (− 131, 347)	86 (− 150, 322)	94 (− 161, 348)	14 (− 206, 233)	287* (52, 523)	269* (47, 491)
Work disability benefits								
All	381** (118, 643)	362** (94, 629)	46 (− 328, 419)	− 51 (− 361, 259)	360 (− 36, 756)	361 (− 86, 808)	515** (166, 865)	487** (134, 839)
Public assistance								
All	356*** (225, 487)	346*** (214, 477)	325** (94, 556)	311** (81, 541)	336* (76, 597)	315* (53, 577)	377*** (206, 548)	371*** (199, 543)
Overall payments combined								
All	938*** (551, 1326)	881*** (491, 1270)	491 (− 40, 1022)	379 (− 135, 893)	793* (176, 1410)	714* (76, 1353)	1172*** (673, 1670)	1124*** (626, 1622)

^*^p < 0.05, **p < 0.01, ***p < 0.001

^a^Including subthreshold depression, major depressive disorder, and persistent depressive disorder

^b^Adjusted for gender and parental education

Moderation analysis revealed a group-by-gender interaction with regard to unemployment benefits. Stratified analysis showed increased levels of unemployment benefits among the depressed males (705 USD, 95% CI 235–1175), as compared with the nondepressed counterparts, while no such pattern was seen among the females (67 USD, 95% CI − 125 to 260) (see Supplementary Table S1). However, there was no evidence of such interactions with other outcomes, nor was gender a significant predictor in other analyses.

### Associations of comorbid and noncomorbid adolescent depression with social transfer payments across adulthood

Depressed adolescents had significantly higher recipiency of overall payments than nondepressed controls, regardless of comorbid/noncomorbid psychopathology (Table 4). Compared with nondepressed controls, those with both comorbid DBDs and ADs evidenced higher recipiency across all specific outcomes, including recipiency of unemployment benefits (566 USD, 95% CI 60–1071), work disability benefits (545 USD, 95% CI 9–1081), and public assistance (634 USD, 95% CI 237–1031). Aside from overall payments, those with only comorbid DBDs also had higher recipiency of public assistance (582 USD, 95% CI 230–935), whereas those with only comorbid ADs also had higher recipiency of work disability benefits (495 USD, 95% CI 67–924). Those with noncomorbid adolescent depression evidenced higher recipiency only regarding public assistance (390 USD, 95% CI 186–594). Most associations remained unchanged after covariate adjustment, and gender was not a significant predictor in any of the analyses.

**Table 4:** Associations of noncomorbid and comorbid adolescent depression with social transfer payments across ages 18–40, with nondepressed controls (*n* = 218) as reference

	Adolescent depression^a^ with noncomorbid and comorbid psychiatric disorders of childhood/adolescence (*n* = 321)							
	Noncomorbid depression (*n* = 132)		Depression + Anxiety disorders (*n* = 93)		Depression + Disruptive behavior disorders (*n* = 44)		Depression + Disruptive behavior disorders + Anxiety disorders (*n* = 52)	
Social transfer payments (USD/year)	Unadjusted	Adjusted^b^	Unadjusted	Adjusted^b^	Unadjusted	Adjusted^b^	Unadjusted	Adjusted^b^
	B (95% CI)	B (95% CI)	B (95% CI)	B (95% CI)	B (95% CI)	B (95% CI)	B (95% CI)	B (95% CI)
Unemployment benefits								
All	82 (− 118, 282)	36 (− 149, 221)	70 (− 143, 283)	79 (− 140, 297)	441 (− 19, 900)	453 (− 66, 972)	566* (60, 1071)	534* (90, 977)
Work disability benefits								
All	291 (− 71, 653)	228 (− 122, 578)	495* (67, 924)	466* (39, 893)	199 (− 255, 653)	155 (− 354, 663)	545* (9, 1081)	565 (− 24, 1153)
Public assistance								
All	390*** (186, 594)	377*** (174, 580)	55 (− 51, 162)	52 (− 48, 152)	582*** (230, 935)	552** (202, 902)	634** (237, 1031)	638** (234, 1042)
Overall payments combined								
All	760** (236, 1285)	624* (120, 1128)	614* (81, 1147)	575* (48, 1103)	1231** (447, 2016)	1152** (306, 1998)	1753*** (887, 2620)	1772*** (866, 2678)

^*^p < 0.05, **p < 0.01, ***p < 0.001

^a^Including subthreshold depression, major depressive disorder, and persistent depressive disorder

^b^Adjusted for gender and parental education

### Associations of adolescent depressive disorders and early psychiatric disorders with overall payments across adulthood

Unadjusted analysis revealed that PDD (1058 USD, 95% CI 529–1588) and MDD (711 USD, 95% CI 67–1356), as well as DBDs (979 USD, 95% CI 344–1614), were associated with higher recipiency of overall payments (Table 5). These associations remained roughly unchanged after covariate adjustment. Further, there was a nonsignificant association between subthreshold depression and overall payments. This was also noted for those with anxiety, albeit in the form of an inverse relationship.

**Table 5:** Associations of adolescent depressive disorders and early psychiatric disorders with overall social transfer payments across ages 18–40

	Overall payments combined (USD/year)	
	Unadjusted	Adjusted^a^
	B (95% CI)	B (95% CI)
Subthreshold depression	305 (− 231, 840)	205 (− 314, 724)
Major depressive disorder	711* (67, 1356)	660* (8, 1311)
Persistent depressive disorder	1058*** (529, 1588)	1006*** (515, 1497)
Disruptive behavior disorders	979** (344, 1614)	961** (362, 1559)
Anxiety disorders	− 215 (− 633, 203)	− 250 (− 632, 132)

^*^p < 0.05, **p < 0.01, ***p < 0.001

^a^Adjusted for gender and parental education

Furthermore, most analyses focusing on age-specific periods in adulthood showed that adolescent depression was consistently associated with higher recipiency of overall payments between groups and across time, particularly for those with PDD or comorbid DBDs, as compared with the nondepressed controls (see Supplementary Tables S2–S4).

## Discussion

This study is the first to quantify the magnitude of the association between depression in adolescence and social transfer payments in adulthood. The results presented herein advance our knowledge about the long-term societal consequences of this common mental disorder, including the impact of episode duration and early comorbid psychopathology.

First, this study provides evidence of robust associations between adolescent depression and several domains of social transfer payments across early-to-middle adulthood, with average payments associated with adolescent depression amounting to a total of around 1000 USD per year for each affected individual. Second, there was some evidence of disorder-specific patterns. Those presenting with PDD clearly evidenced a particularly high recipiency across all outcomes, suggesting that this particular subgroup may have driven a substantial part of the differences observed between those with and without adolescent depression. There was also some evidence of elevated levels of payment recipiency among those presenting with MDD and subthreshold depression, although the overall pattern of findings was less clear for these subgroups. Third, there is evidence to suggest that comorbid psychopathology in adolescent depression may play a key role across various welfare sector costs, but also that noncomorbid depression seems to forecast higher public expenditures in certain respects. Fourth, adolescent depressive disorders and DBDs were independently associated with markedly higher overall payments, even when adjusting for family socioeconomic background, further underscoring the societal challenges posed by these conditions in the long run.

There is growing concern that granting long-term benefits to young people with mental illness may trap them in inactivity and poverty, further perpetuating the sequela of welfare dependency [13, 59, 60]. While the underlying mechanisms and causative pathways of adolescent depression are far from clear, there is the possibility that the longer-term negative economic consequences arise from the stagnating effect of early depression on human capital development [9, 26]. Exposure to multiple or chronic episodes of depression early in life may have a particularly detrimental impact on the acquisition of important skills and abilities required for adult functioning [27]. This stagnation may increase the risk of future labor market marginalization, as mirrored in a rising welfare burden over time.

It is noteworthy that the majority of the cases in the ULADS presented with PDD, which arguably distinguishes this cohort from several observational cohorts in this field [42]; however, long episodes of early depression—of about 1 year’s duration—have been reported to be highly prevalent in other similar community-based surveys of adolescent depression [61, 62]. This may suggest that key differences in methodology in terms of, for example, assessment measures and time frames assessed, likely account for many of the discrepant findings in prior research on adolescent depression and, in effect, its heterogeneous long-term outcomes [8, 9]. However, despite the notion that PDD may often be unrecognized both in epidemiological research and clinical practice [8, 63], it still remains unclear how prevalent the depressive disorders (including PDD) are at various ages and developmental stages (i.e., pre/post puberty), and across various social contexts. More rigorous studies are, therefore, much needed to address these gaps of knowledge.

In view of the findings of this study and the well-documented poor prognosis of adolescent depression [8–10, 23–27], it is of paramount importance to intervene through prevention and early treatment approaches that are both efficacious and cost-effective to avert future ill-health and socioeconomic disadvantage. Preventative measures, such as approaches based on cognitive behavioral therapy, have demonstrated favorable effects [64], which is also the case for early treatment and timely contact with healthcare services [65, 66]. However, depressed adolescents with high levels of oppositionality are recognized as a difficult-to-treat subgroup [67]. Nonetheless, it is likely to be a worthy investment to counteract this negative trend early on through scaling up preventative and early treatment services [64, 68]. Scaling up healthcare services for depression has demonstrated good returns on investment in a scenario-based analysis [69]. To better inform policymaking, and with a view to optimizing the reallocation of the limited societal resources, the impact of scaling up early interventions should ideally be addressed in future research using prospective pragmatic cohort studies.

This study utilized a distinct combination of data from in-depth diagnostic interviews, self-report questionnaires, and nationwide population-based registries, allowing for novel analyses of social transfer payments across early-to-middle adulthood. The integration of over 2 decades of consecutive data, and a high retention rate withal, provides altogether a more comprehensive estimation of the adult welfare burden associated with adolescent depression than previously reported. Few other studies on adolescent depression have included such extensive and objective measures of longer-term economic circumstances. However, our results should be interpreted in the context of some limitations. First, unmeasured factors including genetic vulnerability may account for observed associations, and other forms of residual confounding cannot be ruled out. Second, aspects of sample size, distribution of outcome data, and related power issues did limit the number of covariates to be included in the statistical models. For the same reason, the statistically nonsignificant associations observed for the various subgroups of depressed adolescents should be interpreted with caution, as these results may be attributable to reduced sample size and a resulting lack of power to detect potential differences. Further, the analytic strategy did not permit to examine any potential gender differences linked to the various types of adolescent depression, but only adolescent depression more generally. Third, there was no differentiation between childhood- or adolescence-onset of depression, precluding any further tests of associations predicted by age-of-onset. Fourth, the inherent structure of the diagnostic instrument did not permit to disentangle the timing of early psychiatric disorders. Fifth, this study was not without attrition. Still, around 90% of the original cohort was retained for this 25-year follow-up. Sixth, national regulations of social insurance systems have undergone several changes in recent decades, including both minor and major reforms aimed at reducing overall rates of, for example, disability pension. This may have impacted on the magnitude of the observed differences. Finally, the generalizability of findings might be restricted by the social and historical context relevant to this particular cohort.

## Conclusion

The welfare burden of adolescent depressive disorders amounts to considerable public expenditures across early-to-middle adulthood, most notably for those presenting with early chronic/persistent depression or comorbid psychopathology. This emphasizes the need for efficacious prevention and early treatment. However, the societal costing of such interventions should weigh potential returns on investment in various welfare sectors across the lifespan.

## Supplementary Information

Below is the link to the electronic supplementary material.

Supplementary file1 (PDF 183 KB)

## Data Availability

National regulations regarding data retrieved from the Swedish registries prevent us from sharing any data openly, due to reasons related to confidentiality and protection of human privacy.
