# Supplementary material for: Adolescent depression, early psychiatric comorbidities, and adulthood welfare burden: a 25-year longitudinal cohort study
Source: Soc Psychiatry Psychiatr Epidemiol. 2021 Mar 14;56(11):1993–2004. doi: 10.1007/s00127-021-02056-2 (PMC8519903; doi:10.1007/s00127-021-02056-2)
Supplement: Supplementary file 1 — Supplementary file1 (PDF 183 KB) [file 127_2021_2056_MOESM1_ESM.pdf]

Online Supplementary Material

Adolescent depression, early psychiatric comorbidities, and adulthood welfare burden: A 25-year longitudinal cohort study

Iman Alaie,[1] Richard Ssegonja,[2] Anna Philipson,[3] Anne-Liis von Knorring,[1] Margareta Möller,[3] Lars von Knorring,[4] Mia Ramklint,[1] Hannes Bohman,[1] Inna Feldman,[2] Lars Hagberg,[3] Ulf Jonsson[1,5]

[1] Department of Neuroscience, Child and Adolescent Psychiatry, Uppsala University, Uppsala, Sweden
[2] Department of Public Health and Caring Sciences, Child Health and Parenting (CHAP), Uppsala University, Uppsala, Sweden
[3] University Health Care Research Center, Faculty of Medicine and Health, Örebro University, Örebro, Sweden
[4] Department of Neuroscience, Psychiatry, Uppsala University, Uppsala, Sweden
[5] Karolinska Institutet Center of Neurodevelopmental Disorders (KIND), Centre for Psychiatry Research, Department of Women's and Children's Health, Karolinska Institutet, & Stockholm Health Care Services, Stockholm County Council, Stockholm, Sweden

Table S1 Gender-stratified associations of adolescent depression with social transfer payments **Page 2**

Table S2 Associations of adolescent depression with overall transfer payments across age-specific periods **Page 3**

Table S3 Associations of specific types of adolescent depression with overall transfer payments across age-specific periods **Page 4**

Table S4 Associations of noncomorbid and comorbid adolescent depression with overall transfer payments across age-specific periods **Page 5**

Figure S1 Flow chart of study design **Page 6**

Table S1 Gender-stratified associations of adolescent depression with social transfer payments across ages 18 to 40, with nondepressed controls as reference

	Adolescent depression (n=321)	
**Social transfer payments (USD/year)**	Unadjusted	Adjusted[a]
	B (95% CI)	B (95% CI)
Unemployment benefits		
Female	67 (-125, 260)	57 (-126, 240)
Male	705** (235, 1175)	697** (237, 1156)
Work disability benefits		
Female	453*** (175, 732)	424** (141, 708)
Male	106 (-564, 776)	186 (-354, 725)
Public assistance		
Female	373*** (225, 521)	365*** (215, 514)
Male	296* (16, 575)	296* (23, 568)
Overall payments combined		
Female	899*** (478, 1320)	849*** (436, 1262)
Male	1076* (135, 2018)	1161* (269, 2053)

*p<0.05, **p<0.01, ***p<0.001
[a]adjusted for parental education

Table S2 Associations of adolescent depression with overall transfer payments across age-specific periods in adulthood, with nondepressed controls (n=218) as reference

	**Adolescent depression (n=321)**	
**Social transfer payments (USD/year)**	Unadjusted	Adjusted[a]
	B (95% CI)	B (95% CI)
Overall payments combined		
All		
18-20 years of age	663*** (378, 947)	560*** (311, 809)
21-25 years of age	829*** (408, 1251)	764*** (344, 1183)
26-30 years of age	1160*** (566, 1754)	1115*** (534, 1696)
31-35 years of age	1055*** (526, 1585)	1012*** (479, 1545)
36-40 years of age	1235*** (563, 1907)	1235*** (570, 1901)

*p<0.05, **p<0.01, ***p<0.001
[a]adjusted for gender and parental education

Table S3 Associations of specific types of adolescent depression with overall transfer payments across age-specific periods in adulthood, with nondepressed controls (n=218) as reference

	Type of adolescent depression (n=321)					
	**Subthreshold depression (n=64)**		**Major depressive disorder (n=82)**		**Persistent depressive disorder (n=175)**	
**Social transfer payments (USD/year)**	Unadjusted	Adjusted[a]	Unadjusted	Adjusted[a]	Unadjusted	Adjusted[a]
	B (95% CI)	B (95% CI)	B (95% CI)	B (95% CI)	B (95% CI)	B (95% CI)
Overall payments combined						
All						
18-20 years of age	800** (235, 1365)	622* (129, 1114)	594* (128, 1059)	349 (-33, 732)	644*** (301, 986)	613*** (301, 924)
21-25 years of age	545 (-138, 1228)	410 (-206, 1026)	1045** (336, 1753)	973** (253, 1694)	836*** (321, 1352)	792** (271, 1314)
26-30 years of age	277 (-560, 1114)	270 (-489, 1029)	965* (107, 1823)	891* (9, 1774)	1576*** (793, 2360)	1526*** (766, 2287)
31-35 years of age	429 (-324, 1183)	394 (-364, 1152)	659 (-113, 1432)	573 (-219, 1364)	1455*** (725, 2185)	1407*** (678, 2136)
36-40 years of age	643 (-535, 1821)	602 (-563, 1767)	1136* (3, 2269)	1135 (-2, 2272)	1498*** (661, 2334)	1493*** (664, 2323)

*p<0.05, **p<0.01, ***p<0.001
[a]adjusted for gender and parental education

Table S4 Associations of noncomorbid and comorbid adolescent depression with overall transfer payments across age-specific periods in adulthood, with nondepressed controls (n=218) as reference

	**Adolescent depression with noncomorbid and comorbid psychiatric disorders of childhood/adolescence (n=321)**							
	**Noncomorbid depression (n=132)**		**Depression + Anxiety disorders (n=93)**		**Depression + Disruptive behavior disorders (n=44)**		**Depression + Disruptive behavior disorders + Anxiety disorders (n=52)**	
**Social transfer payments (USD/year)**	Unadjusted	Adjusted[a]	Unadjusted	Adjusted[a]	Unadjusted	Adjusted[a]	Unadjusted	Adjusted[a]
	B (95% CI)	B (95% CI)	B (95% CI)	B (95% CI)	B (95% CI)	B (95% CI)	B (95% CI)	B (95% CI)
Overall payments combined								
All								
18-20 years of age	753*** (342, 1165)	532** (200, 864)	362 (-58, 781)	406 (-16, 827)	759** (193, 1326)	405 (-27, 837)	899** (326, 1473)	963*** (417, 1508)
21-25 years of age	824** (246, 1401)	655* (121, 1190)	368 (-212, 948)	276 (-269, 822)	959* (88, 1829)	870 (-126, 1865)	1571*** (666, 2476)	1713*** (736, 2689)
26-30 years of age	633 (-100, 1366)	488 (-186, 1161)	1388** (432, 2344)	1281** (387, 2175)	1637* (278, 2997)	1624* (152, 3096)	1718** (473, 2963)	1822** (527, 3118)
31-35 years of age	723* (49, 1398)	568 (-55, 1190)	706 (-100, 1511)	633 (-146, 1412)	1880* (362, 3399)	1781* (280, 3283)	1979** (745, 3212)	2088** (742, 3433)
36-40 years of age	1036* (110, 1963)	1034* (120, 1949)	455 (-387, 1298)	450 (-393, 1293)	1698* (120, 3276)	1688* (106, 3269)	2820*** (1169, 4470)	2835*** (1167, 4502)

*p<0.05, **p<0.01, ***p<0.001

[a]adjusted for gender and parental education

Figure S1

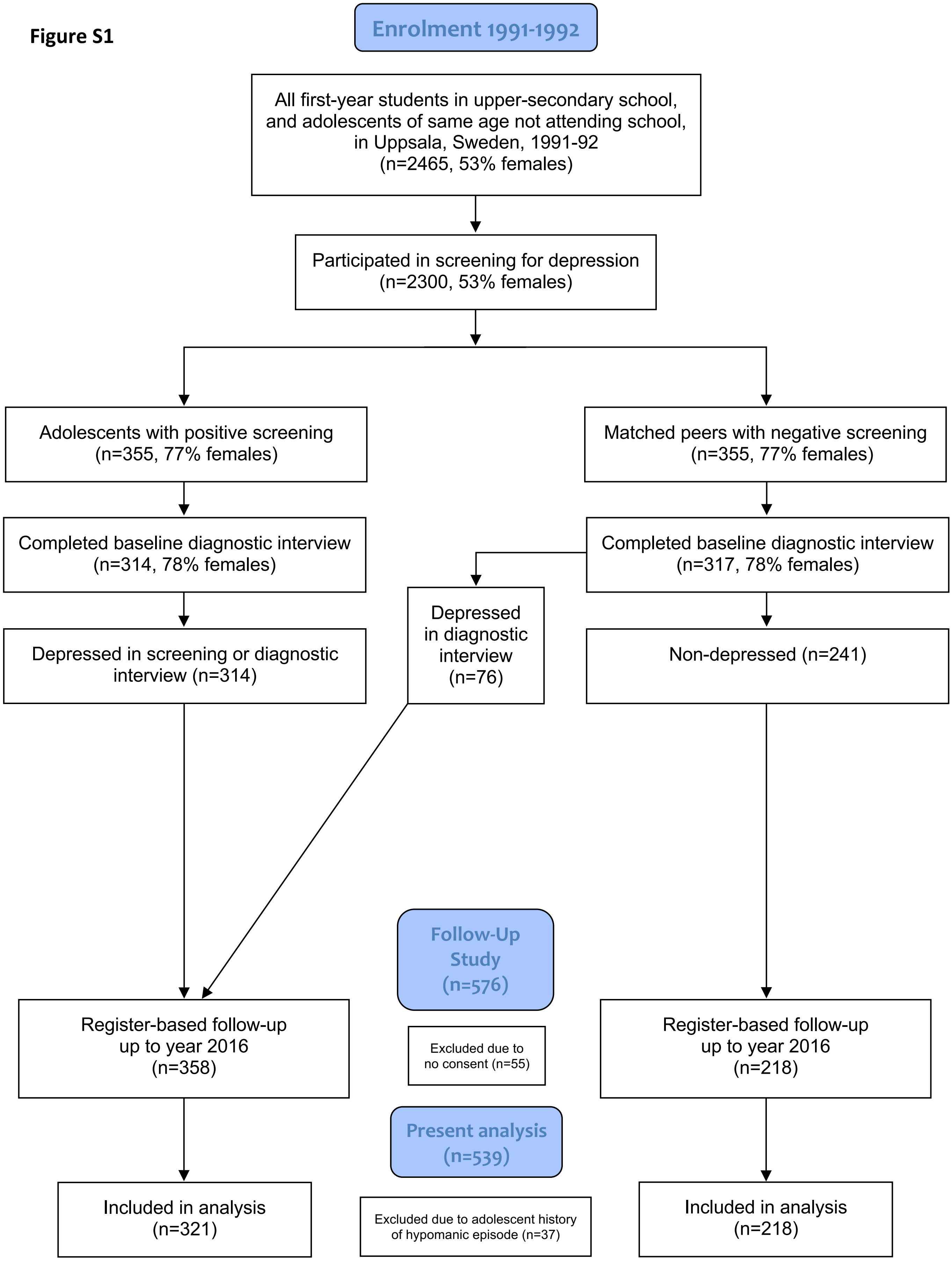
Enrolment 1991-1992
All first-year students in upper-secondary school, and adolescents of same age not attending school, in Uppsala, Sweden, 1991-92 (n=2465, 53% females)
Participated in screening for depression (n=2300, 53% females)
Adolescents with positive screening (n=355, 77% females)
Matched peers with negative screening (n=355, 77% females)
Completed baseline diagnostic interview (n=314, 78% females)
Completed baseline diagnostic interview (n=317, 78% females)
Depressed in screening or diagnostic interview (n=314)
Depressed in diagnostic interview (n=76)
Non-depressed (n=241)
Follow-Up Study (n=576)
Register-based follow-up up to year 2016 (n=358)
Excluded due to no consent (n=55)
Register-based follow-up up to year 2016 (n=218)
Present analysis (n=539)
Included in analysis (n=321)
Excluded due to adolescent history of hypomanic episode (n=37)
Included in analysis (n=218)
